# Identification of Selected Tuna Species in Commercial Products

**DOI:** 10.3390/molecules26041137

**Published:** 2021-02-20

**Authors:** Eliska Servusova, Zora Piskata

**Affiliations:** Depatrment of Infectious Diseases and Preventive Medicine, Veterinary Research Institute, v.v.i., Hudcova 296/70, 62100 Brno, Czech Republic; zorah@email.cz

**Keywords:** *Thunnus albacares*, *Katsuwonus pelamis*, *Sarda* sp., *Auxis* sp., real-time PCR, efficiency, tuna products

## Abstract

This study was conducted to develop systems for the identification of four tuna species (skipjack tuna *Katsuwonus pelamis*, yellowfin tuna *Thunnus albacares*, bullet tuna *Auxis* sp. and Atlantic bonito *Sarda* sp). At first, raw samples of these species and a mix intended as internal control were prepared for the authentication of fish muscle tissue of the genus *Thunnus* sp., *Auxis* sp. and *Sarda* sp. DNA from raw muscle tissue, the mix and samples was extracted with the DNeasy mericon Food Kit (Qiagen GmbH, Hilden, Germany). The concentration and purity of DNA in raw samples were evaluated using a spectrophotometer. Primers and probe sequences were specifically designed to identify the selected species. In addition, primers and a probe for the endogenous *12S rRNA* gene were designed to determine the presence of amplifiable fish (especially tuna) DNA in samples. Furthermore, the species specificity of the designed primers and probes was verified in DNA samples of various tuna and bonito species. Limit of detection for the selected species was calculated as well as the coefficient of determination *R^2^* and efficiency of real-time PCR testing was determined. To evaluate the developed real-time PCR methods, 70 commercial tuna products were analysed. The results show that mislabelling of fish products can still be encountered and, moreover, the presence of an additional species can be identified.

## 1. Introduction

Tuna are among the most popular fish species available on the food market, primarily sold as canned products. The principal species used for canning purposes are skipjack (*Katsuwonus pelamis*) and yellowfin tuna (*Thunnus albacares*). The market also offers raw and frozen fillets, especially made from yellowfin tuna. Different quality and price of various tuna species may lead to a tendency to intentionally or unintentionally substitute different species. Council Regulation (EEC) No. 1536/92 laying down common marketing standards for preserved tuna and bonito, specifies the conditions for tuna marketing. The tuna and bonito species are listed in the Annex to the Regulation. Tuna includes the genus *Thunnus* (*T. thynnus*, *T. albacares*, *T. alalunga*, *T. obesus* and others) and *Euthynnus* and the species *(Katsuwonus) pelamis*. *Sarda* sp., *Euthynnus* sp. (except *Euthynnus pelamis*) and *Auxis* sp. are classified as bonito, known as pseudo-tuna [1]. Pursuant to the Regulation, different species may not be mixed in the same product. The identification of tuna species by morphological features in heavily processed food products is impossible. DNA-based analytical methods provide solution to the problem, even if DNA has been degraded in small fragments during the preservation process, but these fragments are still detectable. Ram et al. (1996) claimed that the canning process degrades DNA to fewer than 123 bp in length [2]. In addition, DNA is largely independent of tissue source, age and sample damage [3,4]. According to DNA-based analysis of tuna species, several studies have described different methods based on multiplex PCR [5,6], real-time PCR [7,8,9,10] and others. For the differentiation between individual fish species on the basis of DNA sequences, the method of DNA sequencing which is still in wide use can be employed for raw samples. Due to the fact that DNA is degraded by thermal treatment during the manufacturing process, a methodology is needed that uses short DNA sequences to distinguish between species. This requirement is met by real-time PCR which allows the identification of DNA sequences consisting of 80 to 200 nucleotides. A major problem in developing a method for distinguishing these fish species based on species-specific DNA sequences is the high identity of DNA sequences among closely related fish species. Regarding canned tuna, most studies preferred mitochondrial DNA to nuclear DNA because of its relative abundance and greater resistance to thermal degradation [3]. There is a close phylogenetic relationship among the *Thunnus* species due to the high homology of their DNA sequences. There is also a relatively high intraspecific variability between tuna species, which makes it difficult to specifically design primers and probes for their identification.

The aim of this study was to develop a method for the identification of four tuna species: yellowfin tuna (*Thunnus albacares*), skipjack tuna (*Katsuwonus pelamis*), Atlantic bonito (*Sarda* sp.) and bullet tuna (*Auxis* sp.) in technologically modified products based on the amplification of species-specific mitochondrial DNA sequences using real-time PCR. To date, no publication has been published on the distinction between preserved tuna and bonito. Most publications focus only on the differentiation of preserved tuna.

## 2. Results and Discussion

### 2.1. Determination of DNA Concentration and Purity

Samples of thawed raw muscle tissue of yellow fin tuna (3), skipjack tuna (3), Atlantic bonito (3) bullet tuna (3) and a mix of *Thunnus* sp. were subjected to the determination of DNA concentration and A260/A280 ratio by a spectrophotometer. The obtained values were averaged and are shown in Table 1. The concentration ranged from 13.5 ng/μL (*S. sarda*) to 36.2 ng/μL (*K. pelamis*). Due to the fact that it is very difficult to get fresh fish for immediate DNA extraction, frozen samples were purchased. This may be one of the reasons for its lower concentration which; however, does not affect further analysis. The ideal value for pure DNA samples should be in the range of 1.7–2.0 [11,12]. Zvarová et al. [13] reported 1.8 as the optimal value. Higher values may indicate the presence of residual protein or phenol, while lower values can indicate very low DNA concentrations.

### 2.2. Specificity

**Figure 1:**
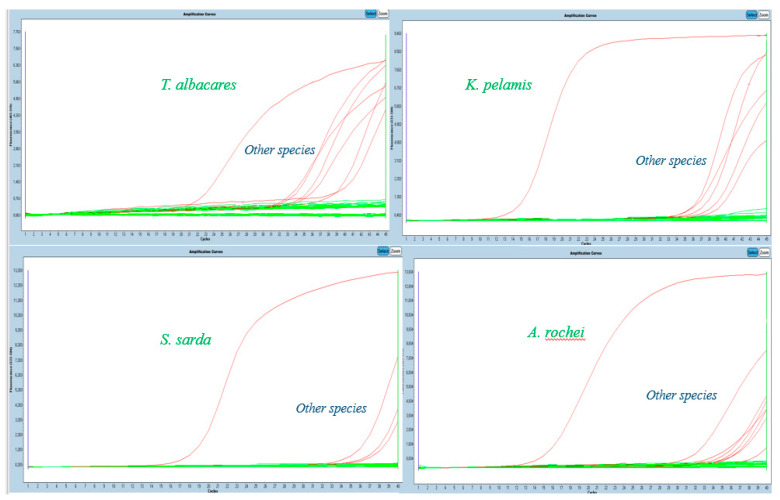
Specificity for different tuna species.

All sequences of total mitochondrial DNA of yellowfin tuna, skipjack tuna and bonitos were compared with all the other mitochondrial DNA sequences of tunas available in GenBank, and species-specific primers and probes were designed to detect yellowfin tuna (84 bp) and skipjack tuna (101 bp), Atlantic bonito (87 bp) and bullet tuna (80 bp). Primers and probes designed for the detection of Atlantic bonito and bullet tuna can also detect other species of *Sarda* and *Auxis* genera and, therefore, they will further be referred to as *Sarda* sp. and *Auxis* sp. For the confirmation of fish DNA (especially tuna presence in samples, mitochondrial *12S rRNA* gene sequence (126 bp) was identified. Besides the in silico comparison of complete mitochondrial DNA sequences of tuna and bonito species, the specificity of the designed primers and probes was verified in DNA samples of the aforementioned tuna species. Based on the primers designed in the present study, we verified the functionality of the systems, because no cross-reactivity between species was observed. Figure 1 shows the analyses of the given species specificity. The study by Chuang et al. [9], dealt with the identification of five true tuna species including cross-specificity with other species. Liu et al. [14] also tested specificity in five true tuna species. No cross-reactivity was demonstrated in any of the studies. Due to the fact that Atlantic bonito is not commonly used for the production of canned tuna but is used exclusively in sashimi and sushi dishes, its specificity is not considered to be an issue [10].

### 2.3. Limit of Detection

Different limits of detection were estimated for each species under defined conditions based on serial dilution of DNA and detection of the Ct value. From the values obtained, the detection limit based on LOD and LOQ (LOD-limit of detection, is defined as the minimum amount or concentration of the analyte in the tested sample, which can be reliably detected; LOQ-limit of quantification, defined as the lowest amount or concentration of the analyte in the tested sample, which can be quantitatively determined with an acceptable degree of accuracy and precision). Table 2 shows that the resulting values are different for each species and that they critically depend on the initial DNA extracted from the sample and its quality. Raw muscle tissue samples were used for serial dilution. However, we usually encounter technologically processed muscle tissue, which can cause inhibition of the subsequent analysis. Due to high temperatures during processing of canned products, which cause DNA destruction, it is relatively difficult to use absolute quantification for DNA assessment. These limits were set on the basis of the requirement for industrially processed products (canning), which makes it possible to determine differences in Ct values between different species [10]. Therefore, close attention should be paid to DNA extraction, especially in processed foods. The most commonly used species for canning purposes is skipjack tuna as follows from both its identification in the canned products and from statistical reports. LOQ were determined to be 0.1 ng/μL for skipjack tuna (with a maximum Ct of 28.55), yellowfin tuna (with a maximum Ct of 33.70) and the genus *Thunnus* sp. (with a maximum Ct of 27.39). Furthermore, LOD and LOQ were 0.01 ng/µL for bullet tuna (with a maximum Ct of 38.86) and 0.001 ng/µL for Atlantic bonito (with a maximum Ct of 32.98). So even though the value for skipjack tuna was determined as 28.55 because of the high severity of stress caused by heat to DNA during manufacturing, we multiplied the value by 1.1 and kept to the Ct value of 31. It follows that, for example, samples of skipjack tuna that had a Ct ˂ 31 were considered positive and vice versa. This fact also applies to the other mentioned species considering their resulting values.

**Figure 2:**
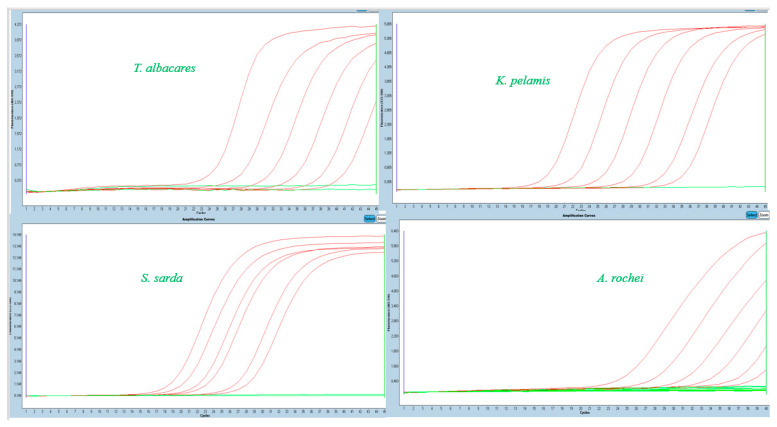
Serial dilution of DNA from different species (10 ng/μL; 1ng/μL; 100 pg/μL; 10 pg/μL; 1pg/μL; 100 fg/μL; 10 fg/μL)—curves from the left according to the indicated dilution.

Figure 2 shows the serial dilution of DNA for each species and the detection of Ct values dependent on fluorescence emission. In earlier studies, Burns and Valdivia [15] suggested Ct 36 to be a cut-off value in modelling the limit of detection in real-time PCR. Rasmussen et al. [16,17] developed a real-time PCR method to identify salmon and trout species based on the cut-off value of Ct ˂ 25 (for fresh and slightly processed samples) and Ct ˂ 30 (for canned samples). Liu et al. [14] in a study dealing with true tunas recognized 30 as a cut-off value of the cycle, and all reactions with Ct > 30 were considered as negative amplification. Chuang et al. [9] set a Ct value at about 16.25 ± 2.65 for yellowfin tuna. Each study is unique, and it is not possible to question the results because many factors can affect the analyses.

### 2.4. Coefficient of Determination R^2^ and Real-Time PCR Efficiency Testing

In the case of real-time PCR performed under the conditions of 100% efficiency, the amplified DNA fragment is doubled in each cycle, which means that the calibration curve has a slope of −3.322, and the shift on the *y*-axis varies under different measurement conditions in relationship to fluorescence measurement sensitivity and fluorescence threshold setting when reading the threshold cycles. In practice, acceptable efficiency for real-time PCR is 90–110% [18], corresponding to a slope of −3.1 to −3.6, where E (efficiency) is determined by the following equation: E = [10^(−1/slope)^ − 1] × 100. The efficiency of the reaction may be influenced by experimental factors (length, secondary structure and GC pair content of the amplicon), dynamics of the reaction, reagent concentration, and quality of the enzyme used. Depending on these factors, the efficiency can decrease below 90%. When PCR inhibitors occur, PCR efficiency is above 110% [14,18].

Even though real-time PCR can be affected by many substances such as polysaccharides, phenolic compounds, proteins and others, the efficiency of PCR for our identification systems ranged from 96.40–107.62%. The efficiency was assessed as 96.40% for yellowfin tuna, 100.42% for skipjack tuna, 107.62% for Atlantic bonito, 102.69% for bullet tuna and 105.58% for *Thunnus* sp. It follows that the range of values was acceptable (90–110%). The efficiency of real-time PCR for five species of tuna in the study of Lui et al. [14] ranged between 90.66% and 102.95% for *T. obesus*, *T. alalunga* and *K. pelamis*, i.e., the values were within the acceptable range. The amplification efficiency for *T. maccoyii* and *T. albacares* in the real-time PCR detection system was 88.05% and 82.20%, respectively. The efficiency of real-time PCR can be reduced due to many factors, such as low ability of probes to bind to their target sequences, dimer or trimer formation between forward primer and reverse primer and probe sequences [14]. The efficiency for canned yellowfin tuna was assessed to be 99.8% ± 5.9% in the study by Bojolly [10]. The study by Terio et al. [19] dealt with mixtures of three tuna species (Atlantic bluefin tuna, yellowfin tuna and albacore tuna) and the slope values ranged from −3.260 to −3.573, and therefore, we can assume that the efficiency was also within the given range. In another study, [10], the efficiency for all systems of five species of true tuna ranged from 102.84% to 120.59%, except for a system using a different probe, in which the values were around 83.11%. The reduced efficiency of the system with the probe could be due to a higher annealing temperature (65 °C) in comparison with the original temperature of 60 °C. Probe and primer sets for valued tuna species are difficult to design as the species are genetically closely related.

**Figure 3:**
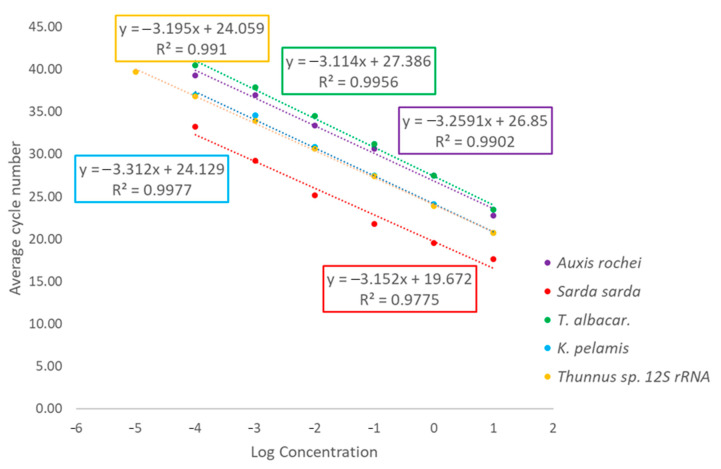
Mean Ct values plotted against the logarithm of the input DNA amount.

Figure 3 shows the Mean Ct values plotted against the logarithm of the input DNA amount.

### 2.5. Verification of the Method in Real Case Samples

A variety of tuna products (*n* = 70) with different compositions were purchased for the analysis. Table 3 shows the names of the products, their specification and type (processing), evaluation of the results and the catch area. The analysis was aimed at authentication of the species declared on the product label. When the obtained results were compared with the species described on the label, it was revealed whether the products were labelled correctly or mislabelled. In addition, we checked if the catch area was or was not specified on the label in compliance with Council Regulation (EC) No. 1536/1992 [1]. Furthermore, the table shows + (correct labelling) or - (mislabelling) for each species and the analysis result giving either the name of the identified species or in case of unidentified species described as unidentified.

**Figure 4:**
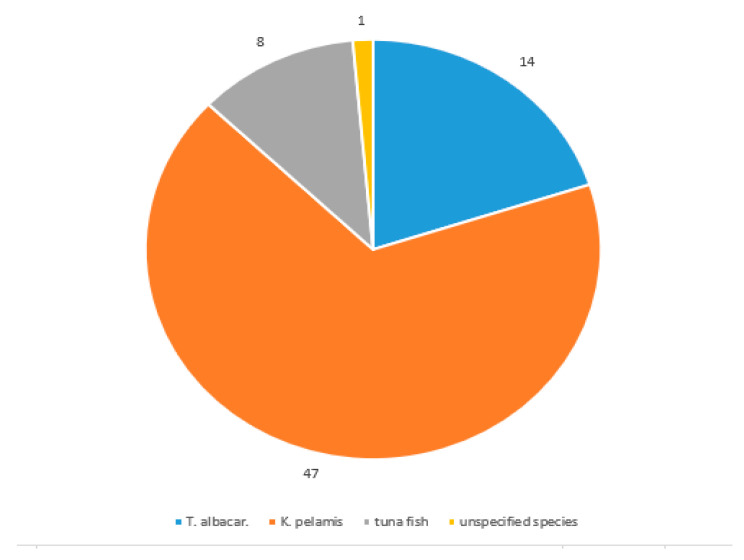
Declaration on the product label.

**Figure 5:**
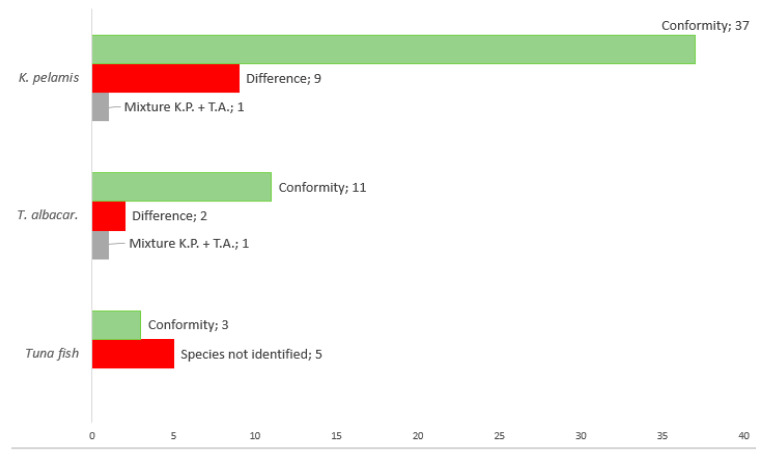
Evaluation of the declaration.

Out of 70 samples, 47 samples were declared as skipjack tuna (67.14%), 14 samples as yellowfin tuna (20%), in 8 cases only the name “tuna” was displayed on the label (11.43%) and in one case no species was specified (1.43%) (Figure 4). When compared with product labelling, the results showed that 38 out of 47 samples (80.85%) were labelled correctly for skipjack tuna; 9 were mislabelled (19.15%), of which in 1 sample (No. 24, *Crushed tuna in its own juice*), yellowfin tuna was also detected, and thus it was probably a mix of the two species. Regarding mislabelling, the samples mentioned below had Ct > 31, and therefore, were designated as mislabelled. These samples included: No. 2—“Tuna in its own juice”, No. 10—“Tuna pieces in sunflower oil”, No. 21—“Tuna chunks in sunflower oil”, No. 35—“Mexico salad”, No. 36—“Texas salad”, No. 45—“Paté”, No. 50—“Salad with cereals”, No. 55—“Tuna with couscous” and No. 56—“Tuna with beans”. Yellowfin tuna was declared in 14 cases, out of which 11 samples (78.57%) were correctly labelled and 3 products were mislabelled (24.43%), with one sample being assessed as a mix of yellowfin tuna and skipjack tuna (No. 62—“Natural Tuna Steaks”) and samples No. 39 (“Tuna Cream”) and No. 67 (“Paté”—“Tuna Paté”—“Paštera od tuna”) were unidentified. In samples displaying only the term “tuna”, skipjack tuna was detected in 3 cases and no species was identified in 5 cases. No species was declared on product No. 3 (“Tuna in its own juice”), but we detected skipjack tuna. Based on the internal amplification control, all samples (*n* = 70) were amplified for fish (tuna) muscle using the *12S rRNA* gene. It follows that tuna was confirmed, but it could be a different tuna species. These results are shown in Figure 5. Surprisingly, no lower-value bonito tuna (*Sarda* sp. or *Auxis* sp.) was detected in any of the samples.

**Figure 6:**
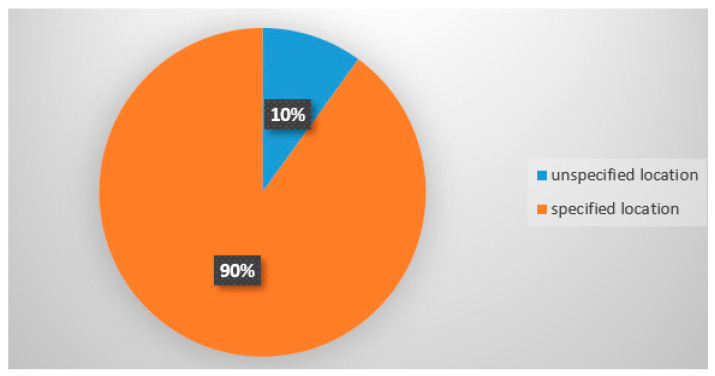
Specification of catch area on the product label.

In compliance with relevant legislation, the product must specify the fishing location or area and the fishing method. Out of 70 samples, 60 products (90%) met the legal requirement for fishing area specification (Figure 6).

Chuang et al. [9], tested six commercial canned products labelled as yellowfin tuna which was confirmed in all of them. Bojolly [10] tested 29 commercial products, of which 11 were labelled as skipjack tuna which was confirmed in all 11 samples, and 10 samples labelled as yellowfin tuna, which were correctly labelled in 5 cases, but in another 5 cases they contained a mix of yellowfin tuna and skipjack tuna. Their results indicated the presence of additional species in yellowfin tuna cans. Mislabelling may also occur during the manufacturing process [10]. The results reported from China indicated a serious safety problem of commercial tuna products as fraudulent labelling was detected in 56% of this type of food [14]. Several methodological strategies have been developed for tuna species identification in raw and preserved products based on the detection of species-specific DNA (PCR-RFLP [7,20], PCR-SSCP [21], PCR-ELISA [22], multiplex PCR [5,6,20] and real-time PCR [8,9,10,21]). DNA barcoding involves PCR analysis followed by sequencing for species identification based on DNA polymorphisms [23]. However, its application may be limited when dealing with fish species identification in products containing a blend of several fish species or in highly processed products where DNA could have been degraded in small fragments that are difficult to detect.

## 3. Materials and Methods

### 3.1. Preparation of Samples

Muscle tissue of yellowfin tuna (*Thunnus albacares*), skipjack tuna (*Katsuwonus pelamis*), Atlantic bonito (*Sarda sarda*) and bullet tuna (*Auxis rochei*) was obtained from markets in the European Union and imported into the Czech Republic. Species identification was carried out according to morphological features in whole pieces of Atlantic bonito and bullet tuna. Yellowfin tuna and skipjack tuna were subjected to sequencing using the *cytochrome b* gene to authenticate species declaration. Furthermore, a mix of fish muscle tissue from different tuna species was prepared as an internal amplification control–*Thunnus* sp. (endogenous gene *12S rRNA*) and was subjected to the same analysis steps. To verify the developed real-time PCR methods, 70 commercial tuna-containing products were analysed. These commercial products were acquired on local markets in the Czech Republic.

### 3.2. DNA Extraction

DNA extraction was performed after careful selection of an appropriate *DNA extraction* procedure (see DNA Extraction section). Out of the analyses considered, the DNeasy mericon Food Kit (Qiagen GmbH, Hilden, Germany) was selected. Thawed tuna muscle tissue samples were processed according to the instructions of the kit manufacturer. Raw muscle analysis was performed in triplicate. Regarding commercial products (*n* = 70), preliminary sample processing was necessary. Analysis of each sample was performed in duplicate. In the case of canned tuna, the muscle tissue was drained in paper towels and freed from possible undesirable ingredients (fat, vegetables, etc.). Pastes, pâtés, spreadable pastes, and samples with thicker sauces were pre-centrifuged. Granules were crushed in a mortar. Total weight taken into the analysis was 200 mg with the addition of lysis buffer and proteinase K under specified conditions (60 °C, 1000 rpm, overnight).

### 3.3. Determination of DNA Concentration and Purity

Raw muscle samples were measured in triplicate using a NanoDrop™ 1000 UV spectrophotometer (Thermo Scientific, Waltham, MA, USA) to obtain DNA concentration and DNA purity based on A260/A280 ratios. The calibration of the instruments was performed using elution buffer. The measurement was performed at room temperature after thorough mixing of all samples. The detected concentrations were further used to perform the dilution series and determine the limit of detection.

### 3.4. Design of Primers and Probes for Real-Time PCR

Primers and probe sequences were specifically designed for the identification of *Thunnus albacares*, *Katsuwonus pelamis*, *Sarda* sp. and *Auxis* sp. DNA sequences of different tuna species were obtained from GenBank (National Center for Biotechnology Information, https://www.ncbi.nlm.nih.gov/nuccore) (accessed on 12 September 2020).

Using Blast (https://blast.ncbi.nlm.nih.gov/Blast.cgi (accessed on 12 September 2020)), all sequences of complete mitochondrial DNA of *Thunnus albacares*, *Katsuwonus pelamis, Sarda* sp. and *Auxis* sp. were compared with all the other mitochondrial DNA sequences of tunas (Table 4) contained in GenBank. The comparison of DNA sequences was performed using the Bioedit (biological sequence alignment editor) program (Carlsbad, CA, USA) to find specific sites for the identification of the species and intraspecific variations. In this study, sections of the *D-loop region* gene were selected to identify the yellowfin tuna, *cytochrome b* gene to identify skipjack tuna, *cytochrome oxidase subunit 1* (COI) gene to identify *Sarda* sp. and *ATPase 6* gene to identify *Auxis* sp. Primers and TaqMan probes were designed and synthesized using the online available Primer 3 software. The probes were labelled at the 5′-end with the fluorescent reporter dye 6-carboxyfluorescein (FAM) for the species *Thunnus albacares* and *Auxis* sp., and at the 3′-end with the BBQ quencher; and at the 5′-end with the dye hexachlorofluorescein (HEX) for species *Katsuwonus pelamis* and *Sarda* sp. and at the 3′-end with the BBQ quencher. In addition, primers and a probe for the endogenous *12S rRNA* gene were designed to determine the presence of amplifiable fish DNA (especially tuna) in samples. The probes for tunas were generally labelled at the 5′-end with the fluorescent reporter dye 6-carboxyfluorescein (FAM) and at the 3′-end labelled with the BBQ quencher. The sequences of primers and probes are shown in Table 5.

### 3.5. Real-Time PCR Conditions

Amplification was performed on the LightCycler 480 II instrument (Roche, Denmark). Based on the investigation which conditions are best suited for the achievement of optimum results, two amplification programmes were set for true tunas and bonitos. For true tunas (*T. albacares* and *K. pelamis*), the following programme was set up: initial denaturation (95 °C for 7 min) for denaturation (95 °C for 10 s), annealing (45 cycles at 60 °C for 15 s), elongation (72 °C for 1 s) and cooling (40 °C, 10 s). The programme for bonitos (*Sarda* sp. and *Auxis* sp.) included: initial denaturation (50 °C, 2 min), denaturation (95 °C, 10 min), annealing (40 cycles for 95 °C, 15 s; 60 °C, 1 min), elongation (72 °C for 1 s) and cooling (40 °C, 10 s). The reaction mixture (20 µL) contained 10 µL of the LightCycler 480 Probes Master kit (Roche, Prague, Czech Republic); 0.2 μL uracil-DNA glycosylase heat-labile (Roche, Prague, Czech Republic); 0.1 μL of each primer (Generi Biotech, Hradec Králové, Czech Republic); 0.05 μL of probe (Generi Biotech, Hradec Králové, Czech Republic); 4.55 μL of PCR H_2_O (Top-Bio, Vestec, Czech Republic) and 5 μL of template DNA. Analysis of each sample was performed in duplicate.

### 3.6. Specificity

Besides in silico specificity testing, species specificity of the designed primers and probes was also verified in DNA samples of the following tuna and bonito species: Frigate tuna (*Auxis thazard*), yellowfin tuna (*Thunnus albacares*), Atlantic bluefin tuna (*Thunnus thynnus*), albacore tuna (*Thunnus alalunga*), bigeye tuna (*Thunnus obesus*), Southern bluefin tuna (*Thunnus maccoyii*), longtail tuna (*Thunnus tonggol*), skipjack tuna (*Katsuwonus pelamis*), Eastern little tuna (*Euthynnus affinis*), Atlantic little tuna (*Euthynnus alletteratus*), and Atlantic bonito (*Sarda sarda*). Furthermore, the specificity was tested in Gadidae and some other sea fish species.

### 3.7. Limit of Detection

The limit of detection was determined on the basis of triplicates of concentrations of DNA extracted from all four above mentioned species, including the genus *Thunnus* sp. The concentrations were measured by a UV spectrophotometer (NanoDropTM 1000, Thermo Scientific, Waltham, MA, USA). The detected concentration for each species was averaged and diluted to the volume of 100 μL in order to obtain the same conditions for each species. For each fish, the DNA concentration was adjusted by serial dilution in water (10 ng/μL; 1ng/μL; 100 pg/μL; 10 pg/μL; 1pg/μL; 100 fg/μL; 10 fg/μL) and the Ct values were determined. Furthermore, the limit of detection (LOD) and the limit of quantification (LOQ) were calculated.

$$\mathrm{LOD}\;=\;\frac{3.3\;\times \;\mathrm{SD}}{b}\;\mathrm{LOQ}\;=\;\frac{10\;\times \;\mathrm{SD}}{b}\;\mathrm{SD}=\mathrm{SE}\;\times \;\sqrt{n}\;\mathrm{Linear}\;\mathrm{regression}\;\mathrm{equation}\;Y=b\;\times \;X+a\;Y=b\;\times \;\mathrm{LOD}+a\;=\;b\;\times \;\left(\frac{3.3\;\times \;\mathrm{SD}}{b}\right)+a\;Y=b\;\times \;\mathrm{LOQ}+a\;=\;b\;\times \;\left(\frac{10\;\times \;\mathrm{SD}}{b}\right)+a$$

SD (standard deviation); SE (standard error of the mean); *n* (numbers of tests); b = slope; a = intercept.

### 3.8. Coefficient of Determination R^2^ and Real-Time PCR Efficiency Testing

From the obtained data, linear regression and appropriateness of the model, i.e., the coefficient of determination *R^2^*, was calculated. To evaluate the efficiency of the designed real-time PCR systems, triplicates of each species were tested based on serial dilutions. The efficiency was assessed by plotting the Ct values against the logarithm of DNA concentration with the following efficiency:

E = [10^(−1/slope)^ – 1] × 100%

### 3.9. Verification of the Method in Real Case Samples

A wide range (*n* = 70) of tuna products were purchased. They were mostly canned fish in their own juices, oils, enriched with ingredients, as well as pastes, pâtés, different tuna salads and tuna dishes. The samples included three pet food products.

The aim of the analysis was the authentication of the species declared on the product label. The labels were carefully inspected, and the obtained results of the analysis were associated either with correct labelling or mislabelling and plotted in diagrams. In addition, we investigated whether or not the catch area was displayed on the label as required by legislation.

## 4. Conclusions

The real-time PCR systems developed in this study allow us to identify four tuna species (skipjack tuna, yellowfin tuna, bullet tuna and Atlantic bonito) and simultaneously to avoid false-negative results. The identification of these species was based on the development of real-time PCR, the design of in-house made primers and probes for the given species, and moreover, the design of a primer and a probe for internal amplification control to confirm fish muscle tissue in samples. The identification of different tuna species has been the focus of many studies, but none dealt with these species (true bonito vs. false bonito). In addition, a variety of tuna products of different matrices were purchased for testing. The analysis was focused on verification of real-time PCR method and authentication of the species declared in product label. Based on checking the label, we could identify whether the product was labelled correctly or mislabelled. We also checked whether the catch area was specified on the label as required by a valid legislative regulation. The results show that mislabelling of fish products can still be encountered and, moreover, the presence of an additional species can be identified. The comparison of the results obtained in the present study with the species declared on the product label showed that 38 out of 47 (80.85%) samples of skipjack tuna were labelled correctly and 9 were mislabelled (19.15%). Yellowfin tuna was declared in 14 cases, out of which 11 samples (78.57%) were labelled correctly when compared with the product label, and 3 samples were mislabelled (24.43%), with 1 sample being identified as a mix of yellowfin tuna and skipjack tuna. Bonitos were not detected in any of the samples.

The question arises as to whether the substitution is intentional or unintentional. Even though this is no threat to the consumers’ health, they still have the right to information what they are buying and what the product contains pursuant to Act 634/1992 Coll. [24], which unfortunately would not be possible today without labelling regulations.

Generally, species mislabelling can be considered a manufacturer’s mistake rather than intentional deception of the consumer by substitution with a less valuable product (for example, a lower-priced meat or other less valuable species being used as a higher). Due to the intensive thermal process which causes DNA degradation used in canned products, it is relatively difficult to use absolute quantification for DNA content. Therefore, it should be emphasized in this study that highly degraded DNA and the presence of PCR inhibitors in processed food products can interfere with species identification of tunas in foodstuffs.

## Figures and Tables

**Table 1:** Determination of DNA concentration and purity in selected species.

Species	Average Concentration [ng/μL]	A260/A280
*T. albacares*	26.3	2.1
*K. pelamis*	36.2	2.0
*S. sarda*	13.5	1.67
*A. rochei*	17.1	1.71
*Thunnus* sp.	28.5	1.85

**Table 2:** Limit of detection for selected species.

Average Ct Values (y-Axis)					
Dilution (x-Axis)	*Auxis rochei*	*Sarda sarda*	*T. albacar.*	*K. pelamis*	*Thunnus* sp.
10 ng/μL	22.73	17.63	23.47	20.72	20.73
1ng/μL	27.45	19.50	27.50	24.09	23.89
100 pg/μL	30.61	21.75	**31.21**	**27.45**	**27.39**
10 pg/μL	**33.40**	25.12	34.50	30.81	30.62
1 pg/μL	36.96	**29.20**	37.87	34.56	33.93
100 fg/μL	39.28	33.20	40.47	36.95	36.85
10 fg/μL	-	-	-	-	39.73
LOD	29.82	24.06	29.47	25.59	25.14
LOQ	35.86	32.98	33.70	28.55	27.39

**Table 3:** List of examined samples.

Sample	Product Name	Processing	Labelling	K.P.	T.A.	S.S.	A.R.	*12S* *rRNA*	Evaluation	Catch Area
1	Tuna crushed in its own juice	Canning	*K. pelamis*	+	-	-	-	+	*K. pelamis*	Ecuador, FAO 77,87; Pacific O.
2	Tuna chunks in its own juice	Canning	*K. pelamis*	-	-	-	-	+	unspecified	Vietnam FAO 71; Pacific O.
3	Tuna in its own juice	Canning	non-declared	+	-	-	-	*+*	*K. pelamis*	Spain, FAO 34; Atlantic O.
4	Tuna natural	Canning	*K. pelamis*	+	-	-	-	*+*	*K. pelamis*	Mauritius
5	Tuna crushed in its own juice	Canning	*K. pelamis*	+	-	-	-	*+*	*K. pelamis*	Thailand
6	Tuna chunks in its own juice	Canning	*K. pelamis*	+	-	-	-	*+*	*K. pelamis*	Philippines, FAO 71; Pacific O.
7	Tuna chunks in its own juice	Canning	*K. pelamis*	+	-	-	-	*+*	*K. pelamis*	Philippines, FAO 71; Pacific O.
8	Tuna chunks in sunfl. oil	Canning	*K. pelamis*	+	-	-	-	*+*	*K. pelamis*	Italy
9	Tesco tuna chunks in sunfl. oil	Canning	*K. pelamis*	+	-	-	-	*+*	*K. pelamis*	Mauritius, Atlan, Ind. Pacific O.
10	Tesco tuna chunks in sunfl. oil	Canning	*K. pelamis*	-	-	-	-	+	unspecified	Mauritius, Atlan, Ind. Pacific O.
11	Tuna chunks in veget. oil and brine	Canning	*K. pelamis*	+	-	-	-	*+*	*K. pelamis*	Ecuador, FAO 77; 88; Pacific O.
12	Tuna crushed in its own juice	Canning	*K. pelamis*	+	-	-	-	*+*	*K. pelamis*	Vietnam, FAO 71; Pacific O.
13	Tuna steak in sunflower oil	Canning	*T. albacar.*	-	+	-	-	*+*	*T. albacares*	Philippines 1246, FAO 71; 77; Pacific O.
14	Tuna steak in sunflower oil	Canning	Tuna	-	-	-	-	+	unspecified	unspecified
15	Tuna steak in olive oil	Canning	*T. albacar.*	-	+	-	-	*+*	*T. albacares*	unspecified
16	Tuna in tomato	Canning	*K. pelamis*	+	-	-	-	*+*	*K. pelamis*	Spain, FAO 34; Atlantic O.
17	Tuna in olive oil	Canning	*K. pelamis*	+	-	-	-	*+*	*K. pelamis*	Spain, FAO 34; Atlantic O.
18	Tuna in its own juice	Canning	*K. pelamis*	+	-	-	-	*+*	*K. pelamis*	Spain, FAO 34; Atlantic O.
19	Tuna smoked in sunflower oil	Canning	*K. pelamis*	+	-	-	-	*+*	*K. pelamis*	Spain, FAO 34; Atlantic O.
20	Tuna in sunflower oil	Canning	*K. pelamis*	+	-	-	-	*+*	*K. pelamis*	Spain, FAO 34; Atlantic O.
**Sample**										
21	Tuna Chunks in vegetable oil	Canning	*K. pelamis*	-	-	-	-	+	unspecified	Spain, FAO, Atlantic O., Pac., Indian O.
22	Tuna chunks in veget. oil with chili	Canning	*K. pelamis*	+	-	-	-	*+*	*K. pelamis*	Philippines, FAO 71; 77; Pacific O.
23	Tuna steak in sunflower oil	Canning	Tuna	-	-	-	-	+	unspecified	unspecified
24	Tuna crushed in its own juice	Canning	*K. pelamis*	+	+	-	-	+	*K. p*. + *T. a.*	Ecuador, FAO 77; 87 Pacific O.
25	Tuna crushed in brine	Canning	*K. pelamis*	+	-	-	-	*+*	*K. pelamis*	Mautitius, Atl., Ind. Pacific O.
26	Tuna crushed in veget. oil and brine	Canning	*T. albacar.*	-	+	-	-	+	*T. albacares*	Vietnam, FAO 71; Pacific O.
27	Tuna chunks in grew. oil and brine	Canning	*K. pelamis*	+	-	-	-	*+*	*K. pelamis*	Ecuador, FAO 77; 87; Pacific O.
28	Tuna in its own juice	Canning	*K. pelamis*	+	-	-	-	*+*	*K. pelamis*	Spain, FAO 71; 77; 81; 87
29	Tuna in its own juice	Canning	*K. pelamis*	+	-	-	-	*+*	*K. pelamis*	Mauritius, Atlan. Ind., Pacific O.
30	Tuna in olive oil	Canning	*K. pelamis*	+	-	-	-	*+*	*K. pelamis*	Italy
31	Tuna chunks in tomato	Canning	*K. pelamis*	+	-	-	-	*+*	*K. pelamis*	Spain, FAO 27; 31; 34; 41; 47; 51;57; 61; 67; 71; 77; 81; 87
32	Tuna in tomato sauce	Canning	*K. pelamis*	+	-	-	-	*+*	*K. pelamis*	Vietnam, FAO 71; Pacific O.
33	Tuna salad Italiano	Canning	*K. pelamis*	+	-	-	-	*+*	*K. pelamis*	Thailand; FAO T. or Indian O.
34	Tuna salad Mexico	Canning	*K. pelamis*	+	-	-	-	*+*	*K. pelamis*	Thailand, T. or Indian O.
35	Tuna salad Exotic	Canning	*K. pelamis*	-	-	-	-	+	unspecified	Thailand; FAO T. or Indian O.
36	Tuna salad Texas	Canning	*K. pelamis*	-	-	-	-	+	unspecified	Thailand, T. or Indian O.
37	Tuna salad Western	Canning	*K. pelamis*	+	-	-	-	*+*	*K. pelamis*	Thailand, T. or Indian O.
38	Yellowfin tuna steak	Canning	*T. albacar*	-	+	-	-	*+*	*T. albacares*	unspecified
39	Tuna cream	Paté	*T. albacar.*	-	-	-	-	+	unspecified	Thailand, FAO 51; 57; 61; 67; 71; 77; 81; 87
40	Tuna paste	Paste	*K. pelamis*	+	-	-	-	*+*	*K. pelamis*	unspecified
41	Tuna cream Paté de Ton	Paté	*K. pelamis*	+	-	-	-	*+*	*K. pelamis*	Italy
42	Tuna cream with hot peppers	paté	*K. pelamis*	+	-	-	-	*+*	*K. pelamis*	Italy
43	Paté Rustico Tonno e Pomodorini	Spreadable cream	*K. pelamis*	+	-	-	-	*+*	*K. pelamis*	Italy
44	Paté Rustico Tonno e Peperoni Dolci	Spreadable cream	*K. pelamis*	+	-	-	-	*+*	*K. pelamis*	Italy
45	Paté Rustico Tonno e Olive	Spreadable cream	*K. pelamis*	-	-	-	-	+	unspecified	Italy
46	French tuna salad (light lunch)	Salad	Tuna	+	-	-	-	*+*	*K. pelamis*	Portugal
47	Tuna salad Mexico (light lunch)	Salad	Tuna	+	-	-	-	*+*	*K. pelamis*	Portugal
48	Tuna in sauce with onion	Salad	*K. pelamis*	+	-	-	-	*+*	*K. pelamis*	Italy
49	Tuna in sauce with red pepper	Salad	*K. pelamis*	+	-	-	-	*+*	*K. pelamis*	Italy
50	Salad Insalatissime 5 cereals	Salad/Canning	*K. pelamis*	-	-	-	-	+	unspecified	Italy
51	Sheba Delikatesse in Gelee	Pouch for cats	Tuna	-	-	-		+	unspecified	unspecified
52	Gourmet Gold with Tuna	Cat food tuna can	Tuna	-	-	-	-	+	unspecified	unspecified
53	Miao Adult with tuna, beef and vegetables	Granules for cats	Tuna	-	-	-		+	unspecified	Holland
54	Tuna in olive oil with chili pepper	Canning	*K. pelamis*	+	-	-	-	*+*	*K. pelamis*	Italy
55	Insalatissime Couscous and tuna	Salad/Canning	*K. pelamis*	-	-	-	-	+	unspecified	Italy
56	Insalatissime Tuna with beans	Salad/Canning	*K. pelamis*	-	-	-	-	+	unspecified	Italy
57	Insalatissime Tuna with potatoes	Salad/Canning	*K. pelamis*	+	-	-	-	*+*	*K. pelamis*	Italy
58	Insalatissime corn and tuna	Salad/Canning	*K. pelamis*	+	-	-	-	*+*	*K. pelamis*	Italy
59	Pasta with tuna	Salad/Canning	*K. pelamis*	+	-	-	-	*+*	*K. pelamis*	Italy
60	Tuna chunks in its own juice	Canning	*K. pelamis*	+	-	-	-	*+*	*K. pelamis*	Thailand, FAO 71; Pacific O.
61	Slices of tuna in olive oil	Canning	*T. albacar*	-	+	-	-	*+*	*T. albacares*	Italy
62	Natural tuna steaks	Canning	*T. albacar*	+	+	-	-	+	*T. a.+ K. p.*	FAO 71
63	Tuna crushed in its own juice	Canning	*T. albacar*	-	+	-	-	*+*	*T. albacares*	Ecuador
64	Tuna steak in its own juice	Canning	*T. albacar*	-	+	-	-	*+*	*T. albacares*	Vietnam
65	Tuna cream	Cream in a can	*T. albacar*	-	+	-	-	*+*	*T. albacares*	Spain
66	Tuna natural	Canning	*T. albacar*	-	+	-	-	*+*	*T. albacares*	FAO 71
67	Paté Pašteta od tune	Canning	*T. albacar*	-	-	-	-	+	unspecified	Croatia
68	Tuna in olive oil	Canning	*T. albacar*	-	+	-	-	*+*	*T. albacares*	Italy
69	Smoked tuna	Canning	*T. albacar*	-	+	-	-	*+*	*T. albacares*	Spain
70	Tuna chunks in its own juice	Canning	Tuna	+	-	-	-	*+*	*K. pelamis*	Ecuador

Note: + detectable; - undetectable.

**Table 4:** Published complete mitochondrial DNA sequences of tunas and bonitos (GenBank; https://www.ncbi.nlm.nih.gov/nuccore) (accessed on 13 September 2020).

Tuna/Bonito	Complete Mitochondrial DNA (GenBank Sequence ID)
*Katsuwonus pelamis*	KM605252, JN086155, GU256527, AB101290
*Thunnus albacares*	KP259550, KM588080, GU256528
*Thunnus alalunga*	JN086151, KP259549, GU256526, AB101291
*Thunnus tonggol*	HQ425780, JN086154
*Thunnus atlanticus*	KU955344, KM405517, KU955343
*Thunnus orientalis*	KF906721, GU256524, AB185022
*Thunnus thynnus*	JN086149, GU256522, KF906720, AY302574, AB097669, AP006034
*Thunnus obesus*	JN086152, GU256525
*Thunnus maccoyii*	JN086150, GU256523, KF925362
*Auxis rochei*	AB103468, KP259548, KM651784, AB105165, AB103467
*Auxis thazard*	KP259551, AB105447
*Euthynnus affinis*	AP012946, KM651783
*Euthynnus alletteratus*	AB099716
*Sarda orientalis*	AP012949
*Sarda chiliensis*	MH194515 *
*Sarda sarda*	KY176599 *, KJ709601 *, KJ768294 *, KC501201 *, JQ623978 *, DQ835917 *

* *cytochrome oxidase I* gene sequence (full mitochondrial sequence for these two species is unavailable in GenBank).

**Table 5:** Sequences of designed primers and probes.

Species	Primers/Probes	Sequence	Targeted Gene	Amplicon Size
Yellowfin tuna *Thunnus albacares*	Forward	5′- CGAGATTTAAGACCTACCATAACAAC-3′	*D-loop region*	84 bp
	Reverse	5′- TGCGCTTAAATTTACCTGACTT-3′		
	Probe FAM-BHQ1	5′- TCGTCTAAGCCATACCAAGTATCCC-3′		
Skipjack tuna *Katsuwonus pelamis*	Forward	5′- TAGACAACGCCACCCTTACC-3′	*Cytochrome b*	101 bp
	Reverse	5′- CGGTTTCGTGAAGGAATAGG-3′		
	Probe HEX-BHQ1	5′- TCCCCTTCGTCATCGCAGCC-3′		
Bullet tuna *Auxis* sp.	Forward	5′- CTTAACATGGGCCTTGCATT-3′	*ATPasa 6*	80 bp
	Reverse	5′- ACCTAGGGCCTCTGTTGGTT-3′		
	Probe FAM-BHQ1	5′- CCCCCTATGACTCGCTACAG-3′		
Atlantic bonito *Sarda* sp.	Forward	5′- GCTGGCATTACAATGCTCCT-3′	*Cytochrome oxidase I*	87 bp
	Reverse	5′- GCTGGTAAAGGATGGGATCA-3′		
	Probe HEX_BHQ1	5′- TTTTTCGACCCTGCAGGCGG-3′		
Tuna and Bonito	Forward	5′- GAGGGGAAGAAATGGGCTAC-3′	*12s RNA*	126 bp
	Reverse	5′- CACTTCAGAGCCGATTTCAGTGGA-3′		
	Probe FAM-BHQ1	5′- CGAATACGAACGATGCACTG-3′		

## Data Availability

Data available in a publicly accessible repository.
